# A Four-Week-Old Infant With Respiratory Distress: An Emergency Department Case Presentation of Congenital Lobar Emphysema

**DOI:** 10.7759/cureus.13814

**Published:** 2021-03-10

**Authors:** Kimberly L Moulton, Andrea Fang

**Affiliations:** 1 Emergency Medicine, Stanford University, Stanford, USA

**Keywords:** congenital lobar emphysema, cle, congenital pulmonary lymphangiectasis, cpl, emergency medicine, pediatric emergency medicine, pem, critical care, infant respiratory distress, respiratory distress

## Abstract

Congenital lobar emphysema (CLE) and congenital pulmonary lymphangiectasis (CPL) are rare conditions that are most often identified with prenatal ultrasonography. Occasionally, this disease process is first identified in the emergency department (ED), where the physician should avoid common pitfalls in order to prevent acute decompensation. To the best of our knowledge, there are no prior reports in the emergency medicine literature of CLE or CPL presenting to the ED as undifferentiated respiratory distress in an infant. Here, we describe one such case and then discuss the importance of differentiating these congenital anomalies from more commonly encountered emergency diagnoses, such as pneumothorax and pneumonia. Management differs radically, and the use of chest tubes and positive pressure ventilation in CLE may precipitate acute cardiovascular decompensation.

## Introduction

Congenital lobar emphysema (CLE) is a rare disorder in young infants who may present to the emergency department (ED); appropriate management and avoidance of pitfalls can be life-saving for the affected child. Here, we discuss a case of an infant who presented with respiratory distress and was found to have a chest X-ray with features concerning CLE. Positive pressure ventilation and thoracostomy were both avoided until the patient was transferred to the operating room, where a pediatric surgery team was able to perform emergent thoracotomy after respiratory arrest following induction of anesthesia and intubation. While management of these patients has been described previously in the anesthesia and surgery literature [1,2], there are no case reports in the emergency medicine literature describing an initial presentation to the ED and outlining the appropriate management for emergency physicians (EPs).

## Case presentation

A four-week-old boy born at term via cesarean section for position presented to a local ED with increased work of breathing for three days. The parents noted that symptoms appeared worse when the infant was lying down, although he continued to breastfeed, void, and stool normally. On the day of the presentation, the parents noted increased fussiness and periodic grunting. Initial ED vitals showed tachypnea, tachycardia, and hypoxia to 89% on room air. Physical examination demonstrated absent right-sided breath sounds and increased work of breathing with associated subcostal retractions in an otherwise well-appearing, pink infant.

**Figure 1:**
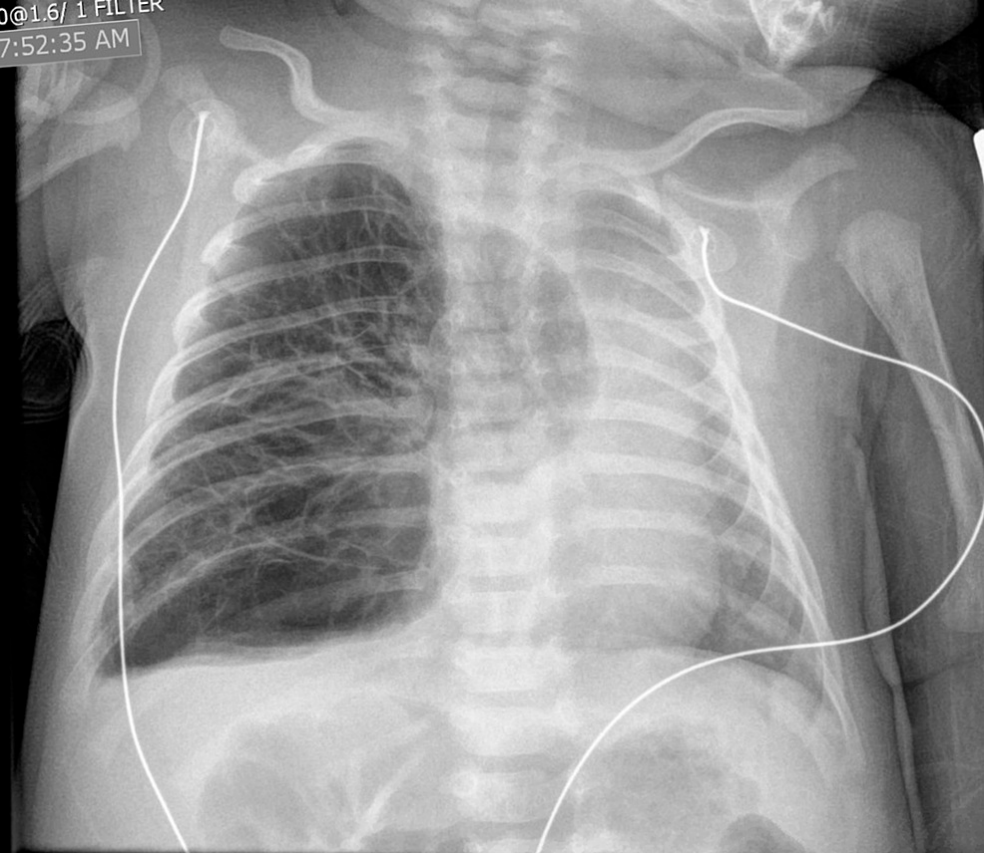
Initial chest X-ray Initial chest X-ray upon presentation. Note the hyperaeration of right-sided lobes, herniation of right lung into the left chest cavity, mediastinal shift, and prominent lung markings within the radiolucent right chest.

A chest X-ray showed right lung hyperinflation with mediastinal shift and herniation of the right lung into the left chest, concerning CLE (Figure 1). According to the family, prenatal workup and ultrasound imaging had revealed no anomalies. The ED workup, including swabs for both respiratory syncytial virus and influenza, was otherwise negative. The infant was started on blow-by oxygen. The transfer was arranged to a local tertiary care center, and he was transported uneventfully.

**Figure 2:**
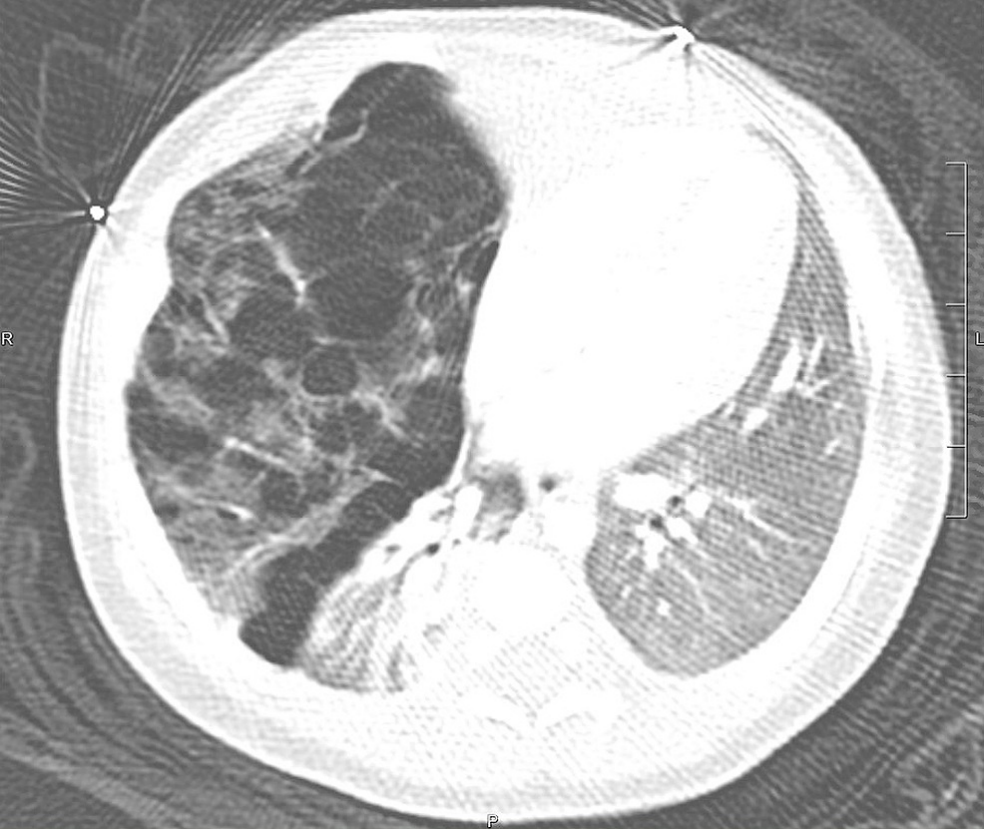
Initial CT Initial CT scan demonstrated emphysematous changes in the right upper and middle lobes, compression atelectasis of the right lower lobe, mediastinal shift, and a relatively normal appearance of the left lung.

Upon admission to the neonatal intensive care unit, a computed tomography (CT) scan of the chest revealed hyperinflation of both the right middle and right upper lobes, with the destruction of normal pulmonary architecture. There was associated mediastinal shift, compression of the right lower lobe with resultant atelectasis, and a normal-appearing left lung (Figure 2).

The infant went to the operating room for urgent thoracotomy with a plan for lobectomy. Anesthesia was induced with glycopyrrolate pretreatment followed by ketamine. Upon intubation, the patient became apneic and was given light positive pressure breaths; however, he became bradycardic as his oxygen saturation continued to decline. Of note, appropriate endotracheal tube positioning was subsequently confirmed with fiberoptic visualization. As the surgical team prepared for emergent incision, he remained severely hypoxic and was given 10 mcg epinephrine (a 2 mcg/kg dose) twice. An incision was made in the fourth intercostal space, and the surgical team noted both pneumothorax and hyperinflation of the right upper and middle lobes. These lobes were delivered outside the chest cavity with the slow recovery of oxygen saturation and heart rate. Lobectomy was performed and the infant tolerated the remainder of the procedure well.

**Figure 3:**
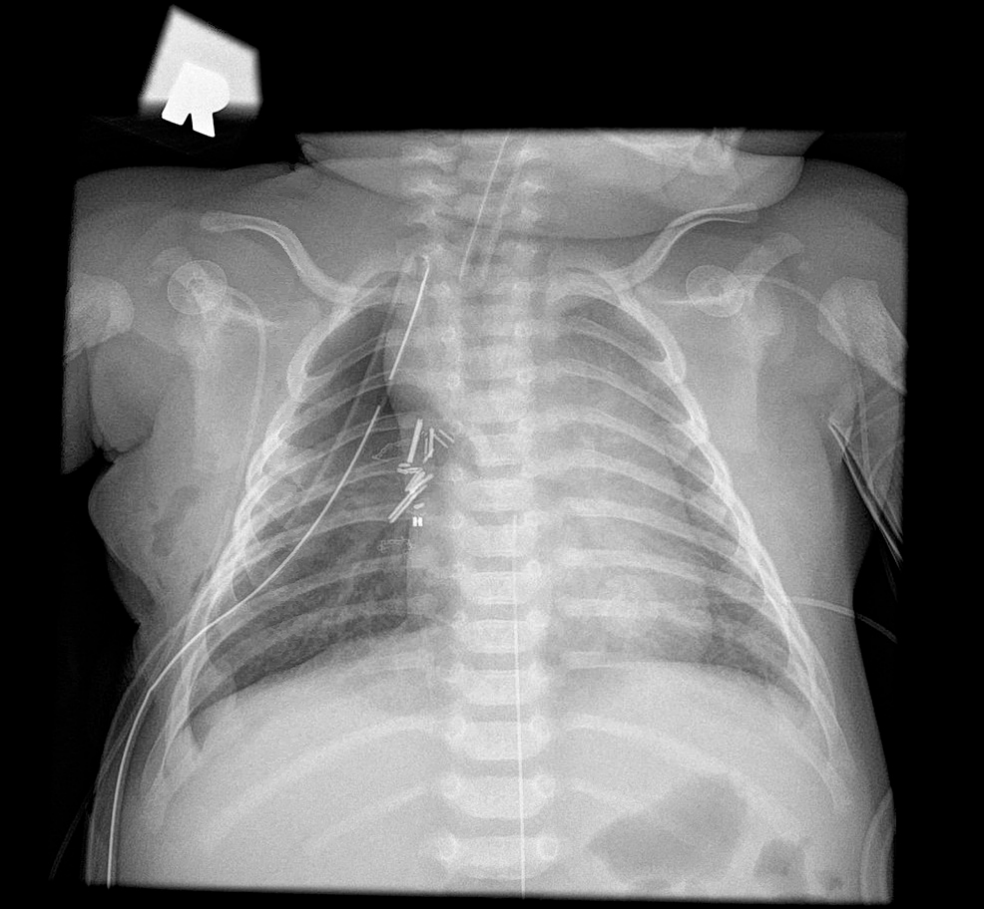
Post-operative X-ray Immediate post-operative X-ray demonstrates resolution of mediastinal shift and a chest tube in place.

**Figure 4:**
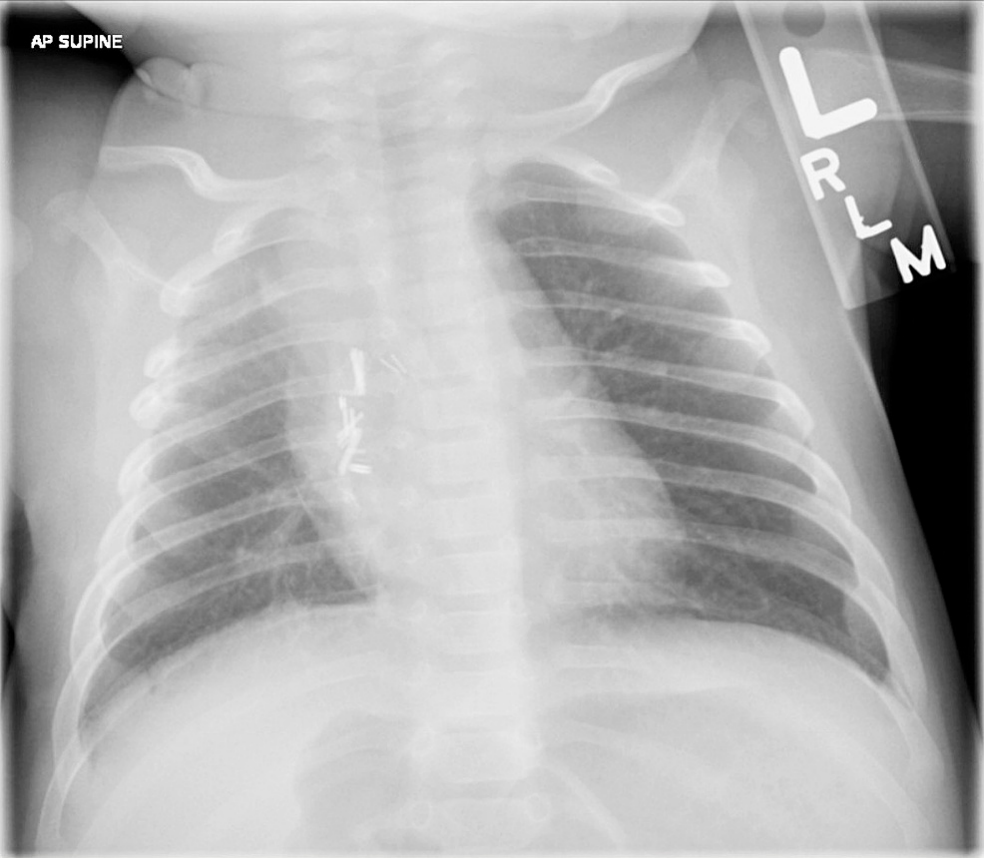
Follow-up X-ray Follow-up imaging three weeks post-operatively shows the appropriate expansion of the right lower lobe.

Immediate post-operative imaging demonstrated resolution of mediastinal shift (Figure 3), and the patient was extubated five hours after returning from the operating room. He continued to recover without complications and was discharged on post-operative day 4. A follow-up chest X-ray demonstrated normal re-expansion of the right lower lobe (Figure 4).

Histologic examination of resected lung tissue performed by a pediatric pathologist demonstrated dilated airspaces, consistent with CLE, and markedly dilated vascular spaces, consistent with congenital pulmonary lymphangiectasia (CPL).

## Discussion

Congenital lobar emphysema is a rare disorder with an estimated incidence of around one in 70,000 live births [1]. While the etiology of CLE varies [3-5], ED management and definitive treatment are the same regardless of the underlying cause [6]. Typically, emphysematous changes affect a single lobe, most commonly the left upper, followed by the right middle and right upper lobe [2,5-7]. Occasionally, multiple lobes are involved [5].

An estimated 95% of CLE cases present clinically in the first six months of life [8]. In the United States, most cases can be identified with routine prenatal ultrasonography [9]. More severe diseases will present earlier, with up to half of patients becoming symptomatic within the first two days of life [6]. The most common presenting symptoms are dyspnea, tachypnea, tachycardia, and cyanosis [10]. Presentations after the first month are typically less severe and may involve recurrent respiratory infections and difficulty with feeding [10,11]. Physical examination typically reveals decreased lung sounds and hyperresonance over the affected side [6,11].

With severe lobar distention, surrounding structures also become compromised [6]. The three key clinical features that explain the presentation of CLE are (1) overdistention and air trapping in the affected lobe(s), (2) compression of healthy lung tissue due to displacement by the affected lobe(s), and (3) compression of mediastinal structures due to displacement by the affected lobe(s), the last of which results in physiology similar to a tension pneumothorax [5]. The natural history of the disease will often lead to worsening hypoxia and ventilation/perfusion mismatch, creating a system vulnerable to cardiopulmonary collapse. This precarious hemodynamic state necessitates caution on the part of EPs caring for these patients, since CLE may mimic diseases that are treated with interventions that increase intrathoracic pressure, thus decreasing venous return and cardiac output and potentially precipitating shock and cardiac arrest [1,6].

**Figure 5:**
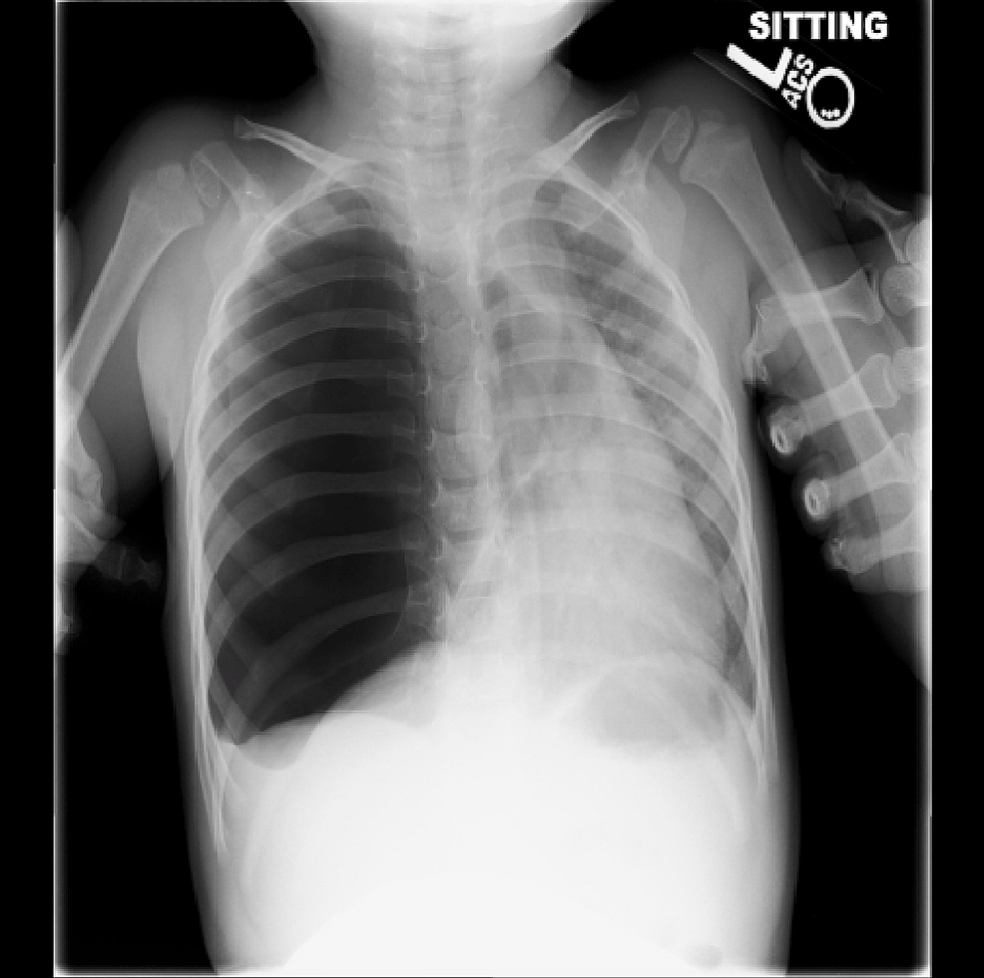
Example pediatric pneumothorax Example chest X-ray showing a massive right-sided pneumothorax with a mediastinal shift in a two-year-old child. Note the absence of right-sided lung markings. Reprinted from Naylor et al. [15] in accordance with the terms of the Creative Commons Attribution License.

The significant challenge to the EP lies in the fact that CLE may mimic much more commonly encountered clinical entities such as pneumonia or pneumothorax [3]. The tracheal deviation is often present on physical examination, further confusing the clinical picture [11]. The distinguishing chest X-ray findings typical for CLE include hyperaeration of the affected lobe, compression of adjacent lobes, mediastinal shift, herniation of lung through to the unaffected side, widened rib interspaces, flattened ipsilateral hemidiaphragm, and sometimes opacity of the affected lobe, especially in the setting of post-obstructive pneumonia [6,12-14]. A lobar pattern of hyperaeration can help to distinguish CLE from pneumothorax, as can the lung markings present throughout the hyperaerated region [2,4,7]. In contrast, a pneumothorax on chest X-ray should have no lung markings within the radiolucent area where the lung has collapsed (Figure 5).

While there may be only subtle differences in the clinical presentation and radiographic appearance of CLE and pneumothorax, EPs should never place a chest tube or perform a needle decompression in a CLE patient. A properly performed thoracostomy that drains the pleural space does not relieve the underlying problem, since with CLE the problem lies within the lung parenchyma. Since no extra air exists within the pleural cavity, the thoracostomy is likely to result in inadvertent puncture of the lung, and the ensuing iatrogenic pneumothorax may worsen the hemodynamic state of an already vulnerable system, potentially to the point of cardiac arrest [1,4,6,11,16].

CLE patients often present in hypoxic respiratory failure, but the EP must understand that PPV can cause cardiovascular collapse secondary to tension-type physiology caused by the expansion of already-hyperinflated lung lobe(s). If intubation is deemed necessary, low ventilatory pressures should be utilized, and intentional mainstem intubation can be considered in order to achieve single-lung ventilation of the unaffected lung and avoid hyperinflation of the emphysematous lobe [1,5]. For sedation, ketamine is the preferred agent in this clinical setting. Vasodilatory agents are contraindicated as they may precipitate profound hypotension [10]. These patients should be immediately transferred to a center with pediatric surgery for definitive treatment with urgent lobectomy [4,7]. Post-surgical outcomes demonstrate excellent functional recovery [5,6].

The patient in this case report presented with classic signs and symptoms of CLE. Although his mother had apparently received standard prenatal care, no abnormality was detected on antenatal ultrasonography. Initial chest X-ray was highly concerning for CLE, although precise identification of affected lobes on plain film X-ray proved challenging (Figure 1). The involvement of both the right upper and right middle lobes was appreciated on the CT scan (Figure 2). The pathology for this patient demonstrated highly dilated vascular spaces most consistent with CPL, in addition to overdistended alveoli consistent with emphysematous changes [17]. CPL is a pediatric condition so rare that no incidence has been defined, though it is thought to account for approximately 1% of cases of stillbirth and neonatal demise [18]. It most often involves all lung tissue and therefore is nearly uniformly fatal within a few hours of birth [18]; however, there are reports of survival in cases of limited lobar involvement [17,19]. Management of CPL involves supportive care prior to lobectomy [18]. Scant case reports exist in the broad medical literature of a CLE/CPL overlap syndrome that clinically and radiologically is indistinguishable from CLE but on histologic review resembles CPL [17,19]. Since making the precise diagnosis may require histopathology [17,18], the EP is unlikely to make a definitive diagnosis at the time of presentation. Thus, all patients with a presentation concerning CLE must be treated as such, and positive pressure and thoracostomy should be avoided if at all possible.

## Conclusions

Congenital lobar emphysema is a rare cause of respiratory distress and recurrent pneumonia in infants. Distinguishing it from other diseases more commonly encountered in the ED, including pneumothorax and simple pneumonia, is imperative since common treatments such as tube thoracostomy and PPV may precipitate arrest. CLE and other congenital abnormalities must be considered in pediatric patients presenting with atypical presentations of pneumothorax or pneumonia, inconsistent chest X-ray findings, or isolated respiratory symptoms without an infectious or cardiac source, especially in patients who did not undergo standard prenatal care. Identifying lobar emphysematous changes in an infant presenting to the emergency room with respiratory distress will guide appropriate management and lead to avoidance of interventions that may precipitate catastrophic physiologic compromise.
